# Dr. Stone’s Address

**Published:** 1887-10

**Authors:** 


### DR. STONE'S ADDRESS.

We have read with interest and emotion the excellent address of Dr. J. W. Stone, the polite and accomplished representative of the house of Parke, Davis & Co., delivered at Angola, Indiana, before the G. A. R. Post, assembled recently at that city.

The Doctor, after giving a graphic discription of a recent trip through Chattanooga, Birmingham, Atlanta, Macon, and down through the South to the sea, referred eloquently to the siege of Vicksburg and other war incidents.

He alludes to the pleasantness of a home in Atlanta, which he now enjoys. The excellencies of the climate, both in summer and winter, the morality, the patriotism and friendly feeling of the Southern people, especially of the old Confederate soldiers.

One of these who had been reduced from affluence to poverty by the war, and who had been wounded on the same field with the Doctor, and on the opposite side of the contest, is now, through the freemasonry of old soldiership, a warm friend. Of such he found many in the South.

Touched by the fact, he concludes his eloquent address with these remarks: "With hundreds of such friendships as this, can you wonder that I have no patience with bloody-shirt stories or the politicians who manufacture them? We have a new soldier's society, just organizing, "The War Survivors' Union." God bless the man who started it! I am addressing a Grand Army Post. I hope I may live to see it merged some day into a War Survivors' Union, where the old private soldier in gray may sit bv the old soldier in blue, and when that day shall come and the rancor of the great conflict is forgotten, and when our America stands at the head of all nations, a common pride to all her sons. If this magnanimous and paternal Government in the exercise of its paternal function of caring for its unfortunate citizens should extend its kindly hand to my crippled old friend in Middle Georgia, and noting his blighted life, his manly effort in his unaccustomed poverty and toil, furnish him the means to educate and fit his son to be an American citizen, and keep his daughter so that in her true womanhood, she may be the wife of an American citizen, I will say God speed the day, and God speed the help—although the bank president and his army substitute, the politician and the office-hunter may with one accord howl "rebel pensions."

